# *EIF3E::RSPO2* Fusion in Metastatic Pancreatic Ductal Adenocarcinoma: A Clinical Case Report Suggesting a Putative *KRAS*-Independent Molecular Profile

**DOI:** 10.3390/ijms27135679

**Published:** 2026-06-24

**Authors:** José María Sayagués, Mar Abad, Diego Bueno-Sacristán, Magdalena Sancho, María Belen Rivas, María Teresa Alonso-Márquez, Ana María Moreno, Juan Carlos Montero

**Affiliations:** 1 Department of Pathology, Institute of Biomedical Research of Salamanca (IBSAL), University Hospital of Salamanca, 37007 Salamanca, Spain; marabad@usal.es (M.A.); dbuenos@saludcastillayleon.es (D.B.-S.); msanchos@saludcastillayleon.es (M.S.); mbrivas@saludcastillayleon.es (M.B.R.);; 2 Centro de Investigación Biomédica en Red Cáncer (CIBERONC), 28029 Madrid, Spain; 3 Department of Pharmacy, Institute of Biomedical Research of Salamanca (IBSAL), University Hospital of Salamanca, 37007 Salamanca, Spain; ammoreno@saludcastillayleon.es

**Keywords:** PDAC, gene fusion, *EIF3E::RSPO2*, tumor genomics, Wnt/β-catenin pathway, personalized medicine

## Abstract

Pancreatic ductal adenocarcinoma (PDAC) is molecularly characterized by near-universal *KRAS* mutations and recurrent alterations in *TP53*, *CDKN2A*, and *SMAD4*. Gene fusions are exceptionally rare and have not been established as canonical drivers of PDAC. We report a case of metastatic PDAC harboring an *EIF3E::RSPO2* gene fusion in the absence of detectable *KRAS* or other common driver mutations. A 48-year-old female was diagnosed with stage IV PDAC via endoscopic ultrasound-guided fine-needle aspiration (EUS-FNA). Comprehensive molecular profiling using the Oncomine Precision Assay GX5 revealed no pathogenic single-nucleotide variants, indels, or copy number variations. However, an *EIF3E::RSPO2* fusion, predicted to be a gain-of-function alteration, was identified as the sole genomic alteration. Immunohistochemistry showed retained mismatch repair protein expression and preserved SMAD4. Although *RSPO2* fusions have been described in preclinical colorectal cancer models and are well-established activators of the Wnt signaling pathway in this setting, their clinical occurrence in PDAC remains poorly documented. This finding indicates a *KRAS* wild-type tumor with a potential *KRAS*-independent oncogenic mechanism that may involve aberrant Wnt/β-catenin signaling and raises the possibility of a rare, biologically distinct PDAC subset. Comprehensive genomic profiling in advanced PDAC may uncover actionable non-canonical drivers with therapeutic implications.

## 1. Introduction

Pancreatic cancer is a major contributor to cancer-related mortality worldwide and is widely recognized as one of the most aggressive malignancies [1]. Pancreatic ductal adenocarcinoma (PDAC), the most common histological subtype of pancreatic cancer, remains one of the most lethal solid malignancies, with a five-year survival rate of below 10% [2]. Its genomic landscape is remarkably conserved across patients, dominated by activating *KRAS* mutations in more than 90% of cases, followed by alterations in *TP53*, *CDKN2A* and *SMAD4* [3]. This molecular uniformity has contributed to the perception that PDAC lacks targetable diversity.

Nevertheless, a small fraction of PDACs are *KRAS* wild-type (WT), and a subset of these tumors may exhibit *KRAS*-independent oncogenic signaling driven by alternative molecular alterations. Emerging data suggests that these tumors may harbor alternative oncogenic drivers, including gene fusions involving *ALK*, *NTRK*, *RET, NRG1* and *RAF* family members [4].

Current standard treatment for PDAC includes surgical resection when feasible, combined with perioperative chemotherapy, whereas advanced disease is mainly managed with systemic chemotherapy. Nevertheless, only approximately 10% of patients are currently eligible for biomarker-driven targeted therapies, particularly those with homologous recombination deficiency (PARP inhibitors such as olaparib) or *KRAS* WT tumors harboring actionable alterations such as dMMR/MSI-H (pembrolizumab), *BRAF* mutations (dabrafenib plus trametinib), or *NTRK*, *RET*, and *NRG1* fusions (larotrectinib/entrectinib, selpercatinib/pralsetinib, and zenocutuzumab, respectively) [5]. Meanwhile, *KRAS* inhibitors are emerging as promising therapeutic strategies for selected *KRAS*-mutant subtypes; however, most PDAC cases still lack actionable molecular alterations, underscoring the need to identify additional therapeutic targets [5].

R-spondin (RSPO) fusions, particularly involving *RSPO2* and *RSPO3*, have been identified and are well-characterized activators of the Wnt signaling pathway in colorectal carcinoma [6,7,8,9,10,11,12]. Wnt pathway activation has also been implicated in pancreatic tumorigenesis, where ligand-mediated signaling is essential for both initiation and progression. In mouse models, inhibition of Wnt signaling prevents acinar-to-ductal metaplasia and the formation of premalignant PanIN lesions, highlighting its critical role in disease development [13]. Furthermore, monoclonal antibody-mediated inhibition of Wnt signaling targeting frizzled receptors reduces tumor growth and tumorigenicity in orthotopic mouse models, supporting the notion that key oncogenic processes in pancreatic cancer depend on ligand-mediated canonical Wnt signaling [14]. In this context, *RSPO2* fusions may act as potent activators of Wnt signaling in a subset of PDACs, thereby driving aberrant pathway activation. Notably, elevated Wnt activity has been associated with cancer stem-like properties, including enhanced self-renewal, drug resistance, increased metastatic potential, and poorer patient outcomes. Collectively, these findings suggest that *RSPO2*-driven Wnt signaling may represent both a tumor-promoting mechanism and a potential therapeutic vulnerability [15]. In fact, Wnt pathway inhibition has emerged as a potential therapeutic strategy for pancreatic cancer. PORCN inhibitors show strong antiproliferative effects in Wnt ligand-dependent pancreatic cancer models and suppress tumor growth in preclinical studies. In addition, Wnt-targeting agents such as vantictumab and ipafricept have demonstrated antitumor activity and have advanced to early-phase clinical trials [16].

Despite the growing recognition of *KRAS* WT PDAC as a distinct molecular subgroup, its full genomic and biological heterogeneity remains incompletely understood. Recent studies suggest that *KRAS* WT tumors may display unique clinicopathological and molecular features, including enrichment in potentially targetable alterations and distinct patterns of pathway activation. However, many of these alterations are rare and insufficiently characterized, limiting their immediate clinical applicability. In particular, while *RSPO* fusions have been extensively described in colorectal cancer, their presence and functional relevance in pancreatic cancer remain largely unexplored. To date, only limited evidence supports the occurrence of *RSPO*-driven Wnt activation in PDAC, and its therapeutic implications are still unclear. Given the central role of Wnt signaling in pancreatic tumorigenesis and stemness, the identification of *RSPO* fusions could define a biologically distinct and potentially targetable subset of tumors.

Here, we present a metastatic PDAC case harboring an *EIF3E::RSPO2* fusion in the absence of detectable canonical driver mutations, suggesting the existence of a rare *KRAS*-independent molecular subtype potentially driven by aberrant Wnt pathway activation. In this context, the identification and molecular characterization of uncommon, actionable alterations remain critical to expanding therapeutic opportunities in a disease traditionally considered genomically homogeneous and therapeutically intractable.

## 2. Case Presentation

A 48-year-old female (DOB: 11 March 1977) was evaluated in the gastroenterology department (University Hospital of Salamanca, Salamanca, Spain) for a pancreatic mass with radiological evidence of metastatic disease. Endoscopic ultrasound-guided fine-needle aspiration (EUS-FNA) was performed (GF-UCT180; Olympus Corporation, Tokyo, Japan). The specimen was processed as a formalin-fixed paraffin-embedded (FFPE) cell block. Tumor cellularity was estimated at 30%. The tumor was clinically staged as Stage IV at diagnosis. The patient initially presented with abdominal pain, weight loss, and jaundice, which prompted radiological evaluation. Imaging studies revealed a mass in the head of the pancreas with hepatic metastases. The patient’s performance status at diagnosis was ECOG 1.

**Figure 1:**
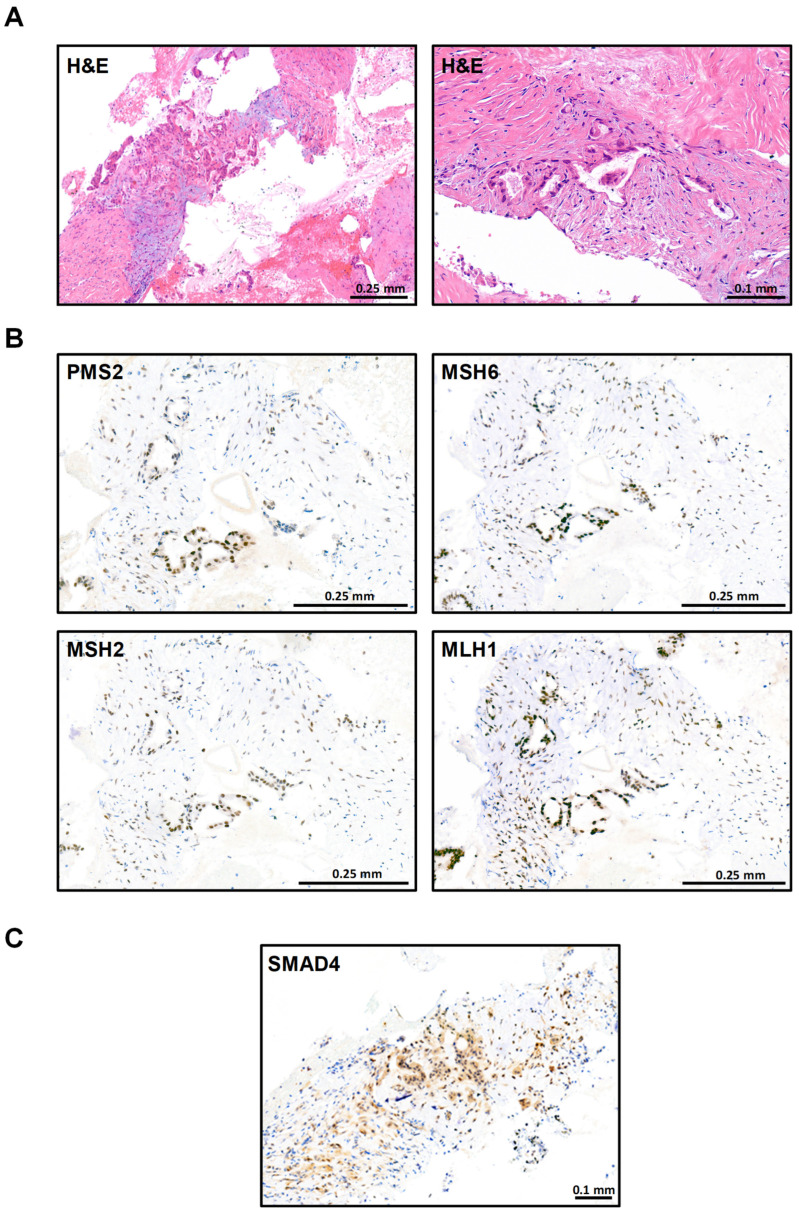
(**A**) Hematoxylin and eosin (H&E) staining of the PDAC sample. Scale bar = 0.25 mm (**left**) and 0.1 mm (**right**). An infiltrative proliferation of irregular angulated glandular structures is observed within a desmoplastic stroma. The neoplastic glands exhibit cytologic atypia, including enlarged, hyperchromatic nuclei and loss of polarity. The surrounding stroma demonstrates a fibro-desmoplastic reaction with mixoid appearance and a mild inflammatory infiltrate. These features are suggestive of ductal adenocarcinoma. Immunohistochemical staining of PMS2, MSH6, MSH2 and MLH1 (**B**) and SMAD4 (**C**) in the PDAC sample. Scale bar = 0.25 mm or 0.1 mm.

Histopathological examination revealed pancreatic ductal adenocarcinoma (PDAC). According to WHO 2022 classification, the lesion was categorized as malignant epithelial neoplasm consistent with ductal adenocarcinoma (Figure 1A). The patient’s tumor sample was analyzed in the Pathology Department of the University Hospital of Salamanca (Salamanca, Spain). For this purpose, 3 µm sections were cut from paraffin-embedded tissue blocks using a microtome and mounted onto glass slides. Immunohistochemical staining was subsequently carried out on a Leica BOND-III automated staining platform using the Bond Polymer Refine Detection system (Leica Biosystems, Newcastle upon Tyne, UK), according to the manufacturer’s instructions. Hematoxylin (Agilent Dako, Gloustrup, Denmark; CS709) and eosin (Agilent Dako, CS711) staining was performed automatically using the Dako CoverStainer (Agilent Dako). Immunohistochemical analysis demonstrated retained expression of MLH1 (prediluted; mouse monoclonal antibody; clone ES05; Leica Biosystems; catalog number PA0988), MSH2 (prediluted; mouse monoclonal antibody; clone 79H11; Leica Biosystems; catalog number PA0989-X), MSH6 (prediluted; mouse monoclonal antibody; clone EP49; Leica Biosystems; catalog number PA0990-X), and PMS2 (prediluted; mouse monoclonal antibody; clone EP51; Leica Biosystems; catalog number PA0991), supporting microsatellite stability (Figure 1B). SMAD4 (DPC4) (rabbit monoclonal antibody; clone EP618Y; Abcam, Cambridge, UK; catalog number ab40759) expression was preserved, excluding loss of this canonical tumor suppressor frequently altered in advanced PDAC (Figure 1C).

The Oncomine Precision Assay (OPA), which enables the simultaneous detection of hotspot mutations (substitutions, insertions and deletions), copy number variations (CNVs), and gene fusions across 50 cancer-related genes using the Ion Torrent GX5 Chip, was used to perform next-generation sequencing (NGS).

To carry out the analysis, three 5 µm sections of the FFPE sample were obtained and deparaffinized. Following tissue lysis, automated nucleic acid extraction was performed using the Genexus™ Purification System (Thermo Fisher Scientific, Waltham, MA, USA). Finally, 20 µL of DNA and RNA (10 ng total input, quantified using a Qubit™ fluorometer) were loaded into a 96-well plate. The plate, containing the nucleic acids and all reagents required for sequencing, was then loaded into the Ion Torrent™ Genexus™ Integrated Sequencer (Thermo Fisher Scientific), a fully automated NGS platform that performs library preparation, template preparation, sequencing, and data analysis within a single workflow. Data were automatically analyzed using the Oncomine^TM^ Reporter software (v.6.1.1) (Thermo Fisher Scientific). The Oncomine Extended filter (version 5.16) was applied for variant calling and annotation. This filter reports hotspot mutations, including amino acids changes and variant allele frequencies (VAFs), of each variant. Furthermore, it indicates the number of readings of detected fusions and provides numerical values for CNVs.

The assay achieved a mean coverage of approximately 2000×, and the panel showed a sensitivity for variant detection of ~3% VAF. Supplementary Table S1 summarizes the 50 genes included in the OPA panel and the types of genomic alterations assessed for each gene.

Molecular analysis revealed no detectable pathogenic or likely pathogenic single-nucleotide variants or indels across the interrogated genes. Importantly, *KRAS* mutational analysis, which covers canonical hotspot codons (12, 13, and 61) as well as additional recurrent non-hotspot regions, including codons 59 and 146, identified no pathogenic *KRAS* alterations across the interrogated regions. Additionally, no CNVs were detected.

**Figure 2:**
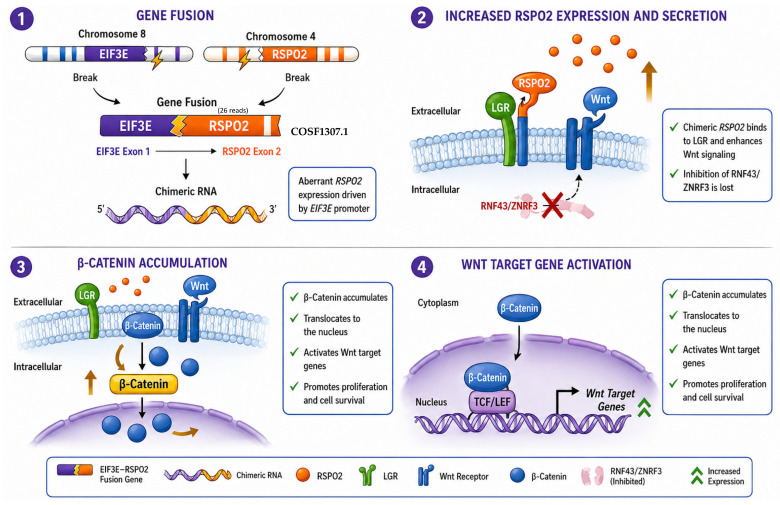
Hypothetical schematic representation of the *EIF3E::RSPO2* gene fusion and its downstream activation of the Wnt/β-catenin pathway. (1) The figure illustrates a chromosomal rearrangement involving chromosome 8, harboring the *EIF3E* gene, and chromosome 4, harboring the *RSPO2* gene. Following chromosomal breaks at both loci, an *EIF3E::RSPO2* gene fusion is generated, in which exon 1 of *EIF3E* is fused to exon 2 of *RSPO2*, resulting in the production of a chimeric mRNA transcript and aberrant *RSPO2* expression driven by the *EIF3E* promoter (variant ID COSF1307.1). (2) Increased expression and secretion of *RSPO2* promote its interaction with LGR receptors at the cell surface, thereby enhancing Wnt signaling. In parallel, the inhibitory activity of RNF43/ZNRF3 is lost, preventing Wnt receptor turnover and further potentiating pathway activation. (3) Sustained Wnt signaling leads to stabilization and intracellular accumulation of β-catenin, which subsequently translocates toward the nucleus. (4) Nuclear β-catenin associates with TCF/LEF transcription factors, resulting in activation of Wnt target genes and increased expression of downstream effectors involved in cell proliferation and survival. Collectively, these events support the proposed oncogenic role of the *EIF3E::RSPO2* fusion through *RSPO2* overexpression and constitutive activation of the Wnt/β-catenin pathway. Figure 2 was generated with the assistance of artificial intelligence for illustrative purposes only.

Fusion analysis identified a single gene rearrangement: *EIF3E* exon 1 fused to *RSPO2* exon 2 (variant ID COSF1307.1) (Figure 2). This rearrangement was predicted to result in a gain-of-function fusion and was supported by 26 sequencing reads. No additional structural alterations were observed. As illustrated in Figure 2, *RSPO2* overexpression is predicted to potentiate Wnt signaling through interaction with LGR receptors, leading to inhibition of the negative regulators RNF43/ZNRF3. This results in stabilization of frizzled receptors at the cell surface and enhanced Wnt pathway activation.

*RSPO2* fusions have been associated with increased transcript expression levels [17]; therefore, we sought to explore whether elevated *RSPO2* expression might have prognostic implications in PDAC. To this end, an in silico survival analysis was performed using the UCSC Xena online database, https://xena.ucsc.edu/ (accessed on 18 May 2026) [18]. Patients were stratified into high- and low-expression groups based on median dichotomization of *RSPO2* expression. From the full series, only patients harboring *KRAS* wild-type tumors were selected and analyzed. Overall survival (OS) was used as the clinical endpoint, and differences between survival curves were assessed using a multivariate log-rank test.

**Figure 3:**
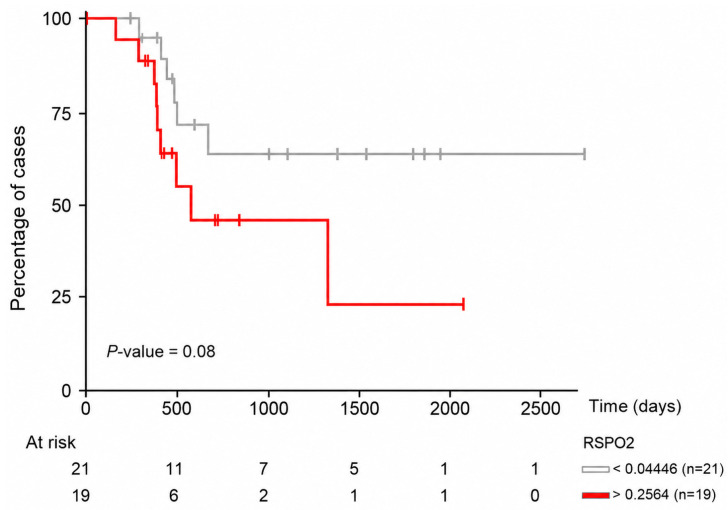
Association between *RSPO2* expression and overall survival (OS) in PDAC. Kaplan–Meier survival curve showing OS in patients from the pancreatic cancer cohort (*n* = 40), stratified by *RSPO2* expression levels. From the full series, only patients harboring *KRAS* wild-type tumors were selected and analyzed. The data were obtained from the UCSC Xena online tool. The *p*-value was calculated and reported by the platform.

Patients with higher *RSPO2* expression exhibited a trend toward reduced OS compared with those with lower expression levels (Figure 3; log-rank *p*= 0.08). However, this difference did not reach statistical significance and should therefore be considered exploratory and hypothesis-generating.

Overall, this case highlights the importance of comprehensive molecular profiling in PDAC, particularly in *KRAS* WT tumors, where rare but potentially actionable alterations such as *RSPO2* fusions may be identified.

## 3. Discussion

Global Burden of Disease 2021 data underscore the growing mortality burden of pancreatic cancer worldwide. Notably, deaths attributable to metabolic risk factors nearly tripled between 1990 and 2021 and are projected to continue increasing through 2050 [19]. This epidemiological context reinforces the urgency of identifying molecularly distinct subgroups, such as the *KRAS* WT PDAC reported here, to guide more precise prevention and treatment strategies.

The *EIF3E* promoter-driven *RSPO2* overexpression predicted by this rearrangement suggests constitutive activation of the Wnt/β-catenin pathway via potentiation of LGR-mediated signaling and inhibition of RNF43/ZNRF3-dependent frizzled receptor degradation [20]. Taken together, the absence of canonical PDAC driver mutations and the presence of a predicted functional *RSPO2* fusion strongly suggest that this rearrangement may represent a probable primary oncogenic driver in this tumor.

This case is remarkable for three reasons. First, the absence of detectable *KRAS* mutation challenges the canonical genomic paradigm of PDAC. While *KRAS* WT PDAC accounts for approximately 5–10% of cases, these tumors frequently harbor alternative actionable drivers [3,5]. In this context, the presence of an *RSPO2* fusion may represent the primary oncogenic driver. Importantly, this case contributes to the emerging concept of *KRAS*-independent PDAC, a biologically distinct subgroup that may rely on alternative signaling pathways for tumor initiation and maintenance. The identification of such cases challenges the long-standing view of PDAC as a uniformly *KRAS*-driven disease and underscores the need for refined molecular stratification. *KRAS* WT PDAC represents a distinct molecular subset frequently driven by actionable gene fusions, including *NTRK*, *ALK*, *NRG1*, *BRAF*, and *FGFR2*, which typically converge on MAPK or *ERBB* signaling pathways. In contrast, the *EIF3E::RSPO2* fusion described here may represent a novel *KRAS*-independent oncogenic mechanism driven by aberrant activation of Wnt/β-catenin signaling, thereby broadening the spectrum of fusion-driven oncogenic pathways in PDAC. However, *RSPO2* fusions have rarely been reported in clinical PDAC specimens. To date, aside from our study, only a single report has identified such events. In that study, a targeted NGS panel (Oncomine Comprehensive Assay v3, Thermo Fisher Scientific) was applied to a cohort of 50 patients with pretreated, advanced metastatic PDAC refractory to standard therapies. Two fusion events were identified: *TBL1XR1::PIK3CA* and *EIF3E::RSPO2*. Notably, these fusions were detected at diagnosis in patients who later developed treatment resistance. However, the study does not provide information on whether these fusions co-occurred with *KRAS* mutations or alterations in other driver genes, leaving open the possibility that they function as primary driver alterations [21].

Second, *RSPO* fusions are established oncogenic drivers in colorectal cancer, where they define a molecular subset mutually exclusive with *APC* mutations and characterized by Wnt pathway hyperactivation [7]. In fact, overexpression of *RSPO2* has been shown to enhance canonical Wnt/β-catenin signaling, which is associated with increased cancer stem cell traits and tumorigenic potential in pancreatic cancer cells. Moreover, *RSPO2*-high subpopulations exhibit greater drug resistance and tumor-initiating capacity, consistent with activation of stemness and survival pathways [15]. Collectively, these findings suggest that *RSPO2* overexpression may contribute to both tumorigenesis and resistance mechanisms, warranting further investigation as a potential therapeutic target in pancreatic cancer. In this regard, Gu et al. recently demonstrated that CDK4/6 inhibition paradoxically activates the Wnt/β-catenin pathway via GSK3β Ser9 phosphorylation, promoting EMT and invasion; combined BET inhibition with JQ1 reversed this effect and synergistically suppressed tumor growth [22]. This convergence suggests that Wnt/β-catenin activation, whether driven by *RSPO2* fusions or pharmacologically induced, operates through shared mechanisms of progression and resistance. In addition, in silico analysis using the UCSC Xena platform demonstrated a trend toward poorer overall survival (OS) in patients with higher *RSPO2* expression, although this association did not reach statistical significance. This finding further supports a potential role for *RSPO2*-driven Wnt signaling in tumor aggressiveness. However, the lack of statistical significance may be attributable to the relatively small sample size of the *KRAS* WT PDAC subgroup (*n* = 40), which may have limited the statistical power to detect significant associations. Nevertheless, additional preclinical studies are required to better define the functional role of *RSPO2* fusions in PDAC and to determine their impact on Wnt pathway dependency. In particular, it will be important to assess whether these aberrations confer specific vulnerabilities that can be therapeutically exploited. Furthermore, prospective clinical studies will be needed to establish the prevalence and clinical relevance of *RSPO2* fusions across different disease stages. Ultimately, such efforts will clarify whether *RSPO2*-driven Wnt activation represents a viable and actionable therapeutic target in this malignancy.

Third, from a therapeutic perspective, this finding may open potential avenues, including: (i) porcupine inhibitors targeting Wnt ligand secretion [23], (ii) LGR5/RSPO axis inhibitors [20] and (iii) Wnt pathway modulators currently in clinical development [24]. Notwithstanding these potential therapeutic implications, it is important to emphasize that Wnt/β-catenin pathway-targeting strategies remain largely investigational and are not currently part of routine clinical management for PDAC. Agents targeting porcupine, the RSPO–LGR axis, or downstream Wnt signaling are still in early-phase clinical development. Therefore, the clinical actionability of *RSPO2* fusions in PDAC should be considered prospective and hypothesis-generating rather than immediately applicable in current practice.

Given the poor prognosis of metastatic PDAC, the identification of rare but targetable molecular subsets is of clear clinical relevance. From a clinical standpoint, these findings reinforce the importance of performing broad molecular profiling in PDAC, particularly in patients with *KRAS* WT tumors, as it may uncover rare alterations with potential biological and therapeutic relevance that would otherwise remain undetected using limited gene panels. Altogether, this case not only expands the molecular spectrum of PDAC but also highlights a potential Wnt-driven oncogenic subset that warrants further investigation in both preclinical and clinical settings. However, as this observation is based on a single case, its generalizability is limited, and larger prospective studies will be required to establish the prevalence, clinical relevance, and distribution of *RSPO2* fusions across different stages of PDAC.

This report has several limitations that should be acknowledged. First, it describes a single case, which limits the generalizability of the findings, as the inclusion of only one patient prevents an understanding of the incidence of this alteration in PDAC. Moreover, it does not allow us to determine whether patients who share this fusion exhibit common clinical, biological, or evolutionary characteristics. Second, independent and functional validation of the *EIF3E::RSPO2* fusion was not performed, and therefore its direct oncogenic role and impact on Wnt pathway activation remain inferential. Future studies should include comprehensive functional characterization of this fusion in *KRAS* WT pancreatic cancer cell line models, including gain- and loss-of-function approaches, to rigorously determine whether it is sufficient to activate Wnt signaling and to promote oncogenic phenotypes such as increased proliferation, survival, and tumorigenic potential. Third, no quantitative assessment of *RSPO2* expression at the transcript or protein level was performed, and therefore the functional impact of the fusion product cannot be directly compared with *RSPO2* overexpression data reported in the literature. Furthermore, immunohistochemical evaluation of β-catenin nuclear localization to confirm canonical Wnt pathway activation could not be carried out due to the limited material obtained from EUS-FNA, precluding additional analyses. Therefore, direct evidence of Wnt/β-catenin pathway activation could not be obtained. Fourth, the NGS analysis was restricted to a panel of 50 cancer-related genes, and for SNVs, only hotspot variants were assessed; therefore, the presence of alternative gene driver alterations in this patient cannot be completely ruled out. In this regard, comprehensive genomic approaches such as whole-exome, whole-genome, or broad hybrid-capture sequencing might identify additional cooperating alterations that were beyond the scope of the present analysis. Fifth, the tumor cellularity of the EUS-FNA specimen was approximately 30%, which is at the lower limit of the recommended range for reliable NGS analysis. This may have reduced the sensitivity for detecting variants present at low variant allele frequencies, and therefore, some low-frequency alterations cannot be fully excluded. Finally, the lack of therapeutic intervention targeting the Wnt pathway in this patient precludes any conclusions regarding the clinical actionability of this alteration. In addition, given the nature of this single-case report and the limitations in the available clinical data, detailed longitudinal follow-up information is not available, and this limitation has been duly acknowledged in the revised manuscript.

## 4. Conclusions

In summary, we report a case of metastatic PDAC harboring an *EIF3E::RSPO2* fusion predicted to confer gain-of-function activity, in the absence of *KRAS* or other canonical driver mutations. This case highlights a rare *KRAS*-independent PDAC harboring an *EIF3E::RSPO2* fusion and raises the possibility that aberrant Wnt pathway activation may contribute to its biology. Comprehensive genomic profiling should be systematically considered in advanced PDAC, particularly in *KRAS* WT cases, to uncover rare but biologically meaningful alterations with potential therapeutic implications.

## Data Availability

The original contributions presented in this study are included in the article/Supplementary Material. Further inquiries can be directed to the corresponding authors.

## Supplementary Materials

The following supporting information can be downloaded at: https://www.mdpi.com/article/10.3390/ijms27135679/s1.
