# Supplementary material for: EIF3E::RSPO2 Fusion in Metastatic Pancreatic Ductal Adenocarcinoma: A Clinical Case Report Suggesting a Putative KRAS-Independent Molecular Profile
Source: Int J Mol Sci. 2026 Jun 24;27(13):5679. doi: 10.3390/ijms27135679 (PMC13362383; doi:10.3390/ijms27135679)
Supplement: Supplementary file 1 [file ijms-27-05679-s001.zip › ijms-4364523-supplementary.pdf]

Supplementary Table S1. Genomic targets included in the Oncomine Precision Assay GX5 panel and alteration types evaluated in the present case.

Alteration type	Genes included in the panel
**Hotspot mutations (SNVs/indels)**	*AKT1, AKT2, AKT3, ALK, AR, ARAF, BRAF, CDK4, CDKN2A, CHEK2, CTNNB1, EGFR, ERBB2, ERBB3, ERBB4, ESR1, FGFR1, FGFR2, FGFR3, FGFR4, FLT3, GNA11, GNAQ, GNAS, HRAS, IDH1, IDH2, KIT, KRAS, MAP2K1, MAP2K2, MET, MTOR, NRAS, NTRK1, NTRK2, NTRK3, PDGFRA, PIK3CA, PTEN, RAF1, RET, ROS1, SMO, TP53*
**Copy Number Variations**	*ALK, AR, CD274, CDKN2A, EGFR, ERBB2, ERBB3, FGFR1, FGFR2, FGFR3, KRAS, MET, PIK3CA, PTEN*
**Intergenic Fusions**	*ALK, BRAF, ESR1, FGFR1, FGFR2, FGFR3, MET, NRG1*
**Intragenic Fusions**	*AR, EGFR, MET, NTRK1, NTRK2, NTRK3, NUTM1, RET, ROS1, RSPO2, RSPO3*
